# Helping Patients With Chronic Conditions Overcome Challenges of High-Deductible Health Plans: Mixed Methods Study

**DOI:** 10.2196/37596

**Published:** 2023-01-31

**Authors:** Tiffany Yung-Shin Hu, Iman Ali, Michele Heisler, Helen Levy, Angela Fagerlin, Jeffrey T Kullgren

**Affiliations:** 1 University of Michigan Ann Arbor, MI United States; 2 Veterans Affairs Ann Arbor Healthcare System Ann Arbor, MI United States; 3 Salt Lake City Veterans Affairs Medical Center Salt Lake City, UT United States; 4 University of Utah Salt Lake City, UT United States

**Keywords:** high-deductible health plan, HDHP, chronic conditions, cost-conscious strategies, consumer behaviors, health care costs, out-of-pocket spending, OOP, behavioral intervention, mobile phone

## Abstract

**Background:**

A growing number of Americans are enrolled in high-deductible health plans (HDHPs). Enrollees in HDHPs, particularly those with chronic conditions, face high out-of-pocket costs and often delay or forgo needed care owing to cost. These challenges could be mitigated by the use of cost-conscious strategies when seeking health care, such as discussing costs with providers, saving for medical expenses, and using web-based tools to compare prices, but few HDHP enrollees engage in such cost-conscious strategies. A novel behavioral intervention could enable HDHP enrollees with chronic conditions to adopt these strategies, but it is unknown which intervention features would be most valued and used by this patient population.

**Objective:**

This study aimed to assess preferences among HDHP enrollees with chronic conditions for a novel behavioral intervention that supports the use of cost-conscious strategies when planning for and seeking health care.

**Methods:**

In an exploratory sequential mixed methods study among HDHP enrollees with chronic conditions, we conducted 20 semistructured telephone interviews and then surveyed 432 participants using a national internet survey panel. Participants were adult HDHP enrollees with diabetes, hypertension, coronary artery disease, chronic obstructive pulmonary disease, or asthma. The interviews and survey assessed participants’ health care experiences when using HDHPs and their preferences for the content, modality, and frequency of use of a novel intervention that would support their use of cost-conscious strategies when seeking health care.

**Results:**

Approximately half (11/20, 55%) of the interview participants reported barriers to using cost-conscious strategies. These included not knowing where to find information and worrying that the use of cost-conscious strategies would be very time consuming. Most (18/20, 90%) interviewees who had discussed costs with providers, saved for medical expenses, or used web-based price comparison tools found these strategies to be helpful for managing their health care costs. Most (17/20, 85%) interviewees expressed interest in an intervention delivered through a website or phone app that would help them compare prices for services at different locations. Survey participants were most interested in learning to compare prices and quality, followed by discussing costs with their providers and putting aside money for care, through a website-based or email-based intervention that they would use a few times a year.

**Conclusions:**

Regular use of cost-conscious strategies could mitigate financial barriers faced by HDHP enrollees with chronic conditions. Interventions to encourage the use of cost-conscious strategies should be delivered through a web-based modality and focus on helping these patients in navigating their HDHPs to better manage their out-of-pocket spending.

## Introduction

### Background

Approximately half of Americans with private health insurance face the risk of high out-of-pocket (OOP) health care spending because they are enrolled in a high-deductible health plan (HDHP), a private health insurance plan with a deductible of at least US $1400 for an individual or US $2800 for a family that can often be combined with a health savings account (HSA) [1]. Growth in HDHP enrollment has increased substantially in recent years, as employers seek to control the growth of health care costs amid rising health insurance premiums [2].

The increase in HDHP enrollment has created financial and access challenges for many patients. The complicated benefit design of HDHPs often leaves enrollees with misunderstandings about covered services and confusion about OOP costs, especially for urgent services [3]. High cost sharing in HDHPs can lead many patients to delay or forgo necessary care [4], including high-priority office visits [5], long-term medications [6], and clinical preventive services, even when such services are exempt from cost sharing [7]. Access to affordable care is particularly challenging for HDHP enrollees with chronic conditions [8,9], who often face substantial financial burdens when enrolled in an HDHP [10,11].

An approach to mitigate these challenges is to help HDHP enrollees with chronic conditions better understand and use their health plans. Use of beneficial cost-conscious strategies can help HDHP enrollees with chronic conditions pay less for health care and have better access to necessary services [12]. Such strategies include discussing costs with providers, which can help patients and providers identify services that the patient will need in the future and determine whether the patient can pursue low-cost care alternatives [13,14]; saving for future health care in an HSA or flexible spending account (FSA) for pretax savings on health care expenses; and using web-based tools to compare price and quality to optimize the value of OOP spending. However, most HDHP enrollees do not routinely use these strategies [15-18], often because they may not consider doing so when planning for and seeking health care [12] or are unaware that such strategies could benefit them [19].

No behavioral intervention has attempted to encourage HDHP enrollees to engage in cost-conscious strategies to reduce needed health care costs, and there are no previous studies on enrollee preferences to guide the design of effective interventions.

### Objective

The objective of this study was to assess the preferences for the content, modality, and frequency of interaction with an intervention to encourage the use of cost-conscious strategies among patients in HDHPs with chronic conditions to inform the development of future novel behavioral interventions.

## Methods

### Ethics Approval

This study was reviewed and approved by the University of Michigan (U-M) Medical School’s institutional review board (HUM00180179). Informed consent was obtained from all participants. To protect participants’ privacy and confidentiality, all study data were deidentified before analysis. Interview participants were compensated with a gift card worth US $25, and survey participants were compensated in accordance with the policies by Dynata for internet survey panel participants.

### Study Design

We conducted an exploratory sequential mixed methods study among HDHP enrollees with chronic conditions. As defined by Creswell and Plano Clark [20], exploratory sequential mixed methods studies use qualitative findings to inform quantitative study methods. For this study, we first conducted 20 semistructured interviews with HDHP enrollees with chronic conditions. Interview findings informed the development of measures for a web-based survey of 432 HDHP enrollees.

### Theoretical Model

**Figure 1:**
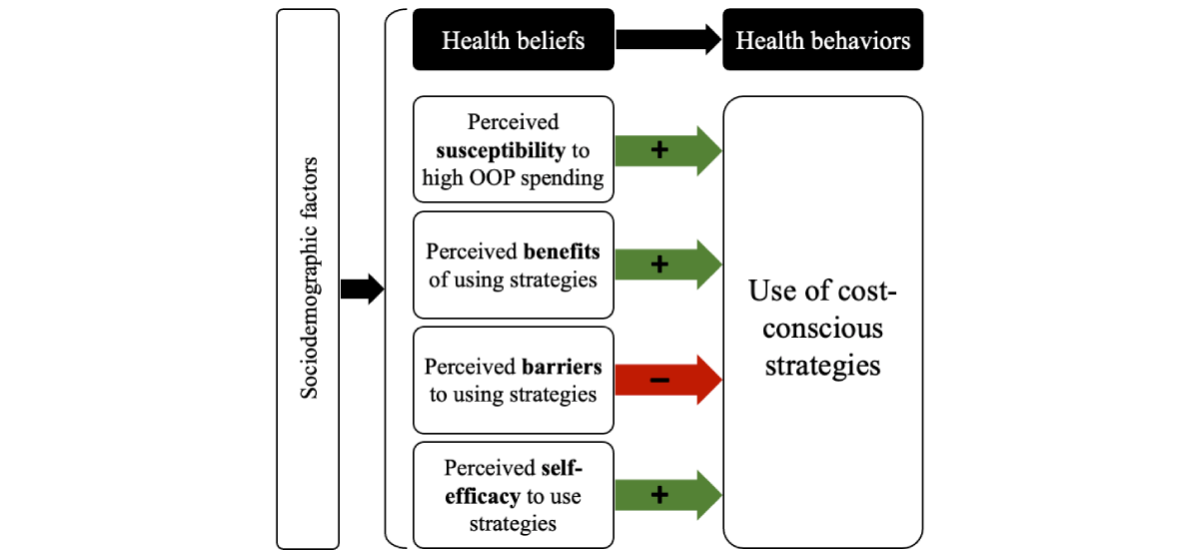
Key constructs of adapted Health Belief Model. OOP: out-of-pocket.

Our semistructured interview guide and survey items were grounded in the conceptualization of cost-conscious strategies as teachable health behaviors. To identify constructs that could facilitate or impede engagement in cost-conscious strategies, we adapted the Health Belief Model [21]. Key constructs in this adaptation included perceived susceptibility to high OOP spending, benefits of and barriers to using cost-conscious strategies, and self-efficacy to engage in cost-conscious strategies (Figure 1). Thus, participants were asked about their previous use of and confidence in engaging in cost-conscious strategies to assess self-efficacy and perceived benefits and barriers. Measures included health service utilization, experiences of delayed and forgone care owing to cost, and participants’ perceived risks of HDHPs (eg, high OOP costs). The Health Belief Model also posits that demographic and structural factors may affect individuals’ beliefs and behaviors. Therefore, data collection included measures of demographic and structural factors hypothesized to be important, such as age, gender, race and ethnicity, income, health literacy and health insurance literacy, and technological access—factors that may influence participants’ perceived risk of high OOP spending, barriers to engaging in cost-conscious strategies, and interest in different intervention components.

### Qualitative Phase—Semistructured Telephone Interviews

From October 2020 to December 2020, we used the U-M Health Research website [22] to recruit 20 adults aged 18 to 65 years, who were enrolled in an HDHP and had at least one of the following chronic conditions: hypertension, asthma, coronary artery disease, and chronic obstructive pulmonary disease. We targeted these common chronic conditions based on our previous study, which showed that these conditions are associated with high OOP spending in HDHPs [10]. Individuals were prescreened for participation through the U-M Health Research website [22] with a questionnaire that asked about their health plan, chronic conditions, and confidence in engaging in cost-conscious strategies. We used purposive sampling to recruit individuals in both employer and exchange plans and those with high and low confidence in their ability to engage in cost-conscious strategies.

Telephone interviews were conducted by a study team member using a semistructured interview guide (refer to the interview guide in Multimedia Appendix 1). Participants were asked to describe their experiences in managing their chronic conditions, selecting and using their HDHPs, and engaging in cost-conscious strategies (eg, discussing costs with providers, saving for future health care, and using web-based tools to compare prices and quality). Participants were then asked about their preferences for the information content, modality, and frequency of use of an intervention to help them manage their health care costs. Interviews were conducted until thematic saturation, which was assessed by rolling rapid analysis of interview notes, was reached. Data on participants’ demographic characteristics, health literacy, health insurance literacy, and confidence in engaging in cost-conscious strategies were collected using survey measures over the phone.

All (20/20, 100%) interviews were audio-recorded, deidentified, transcribed, and analyzed for qualitative themes. A deductive codebook was compiled using the interview guide and systematically applied to all (20/20, 100%) interviews to elicit major themes. Codes and their respective definitions were revised when needed to capture participant’s experiences and preferences. For qualitative analysis, the codebook was uploaded to Dedoose, a cloud-based software platform for qualitative coding. Codes were divided into 4 categories: care for chronic conditions, choice and use of current health plan, cost-conscious strategies in health care, and intervention preferences. Qualitative coding was primarily completed by 2 authors, who consulted with the principal investigator for reconciliation and revisions to the coding system. After revising the codebook to reflect new thematic content, the transcripts were recoded to ensure accuracy. After all the transcripts were double-coded and codes were reconciled, the 2 primary coders conducted thematic analysis to identify overarching themes.

### Quantitative Phase—National Web-Based Survey

On the basis of themes that emerged from interviews, we developed a survey instrument (refer to survey instrument in Multimedia Appendix 1) to assess intervention preferences among a large, nationwide sample of HDHP enrollees with chronic conditions. Table 1 lists the key qualitative themes and the survey items they informed. We collaborated with Dynata (formerly Survey Sampling International), which hosts a national internet survey panel, to survey 432 individuals who had been enrolled in an HDHP for more than a year and had at least one of the previously mentioned common chronic conditions. Survey participants were recruited by Dynata without quotas until the desired sample size was reached, and anyone from their web-based health care panel who met the previously mentioned requirements qualified to complete the survey. The national web-based survey was fielded from January 15, 2021, to January 25, 2021.

Our main survey measures asked participants about their level of interest in different types of potential educational content (ie, discussing costs with providers, saving for future health care, and using web-based tools to compare prices and quality) and different potential modalities for delivering this content (ie, app, website, SMS text messages, emails, mailed documents, peer support, and telephone coaching). Level of interest for both content and modality was measured using a 3-point scale (“Very interested,” “Somewhat interested,” and “Not at all interested”). The 3-point scale for these self-created questions was adopted from Kullgren et al [12] because previous cognitive interviews found that 3-point scales were easy to respond to and sufficient to measure levels of interest. Participants were also asked how frequently they thought they would interact with the intervention.

**Table 1:** Exploratory sequential mixed methods design—interview themes that informed the development of survey measures.

Qualitative themes and categories			Survey items
**Financial and access challenges in HDHP^a^**			
	Delayed and forgone care owing to cost	During the past 12 months... Have you delayed seeking medical care because of worry about the cost? Was there any time when you needed medical care, but did not get it because you couldn’t afford it?	
	Difficulty in affording medical bills	Did you have problems paying or were unable to pay any medical bills?	
**Experiences with cost-conscious strategies**			
	Previous experience in using cost-conscious strategies to manage out-of-pocket spending	In the past 12 months... Did you put aside money to pay for any health care services before you needed them? Did you compare prices for any health services at different places? Did you talk with a health care provider...about how much any health services would cost you personally?	
	Difficulty in knowing whom to talk to about costs	Did you talk with a health care provider...about how much any health services would cost you personally?	
	Low confidence in engaging in cost-conscious strategies	As of right now, how confident are you that you could... Put aside enough money to pay for health care services before you need them? Compare prices for health care services at different places? Talk with a health care provider (e.g. a doctor, nurse, or pharmacist) about the cost for health care services?	
**Key intervention preferences**			
	Content focused on helping patients use cost-conscious strategies	How interested would you be in learning more about... How to put aside money to pay for health care services? Ways to talk to someone at your doctor’s office about your health care costs? Strategies for comparing prices for health care services? Strategies for comparing quality ratings for health care services?	
	Easily accessible technological intervention, phone calls, printed information, and support groups to learn from other patients	How interested would you be in receiving information on these topics through... An app for your smartphone or tablet? A website? Periodic text messages? Periodic emails? Phone coaching sessions? Print materials mailed to you? Tips from other patients?	

^a^HDHP: high-deductible health plan.

In addition, the survey assessed participants’ use of cost-conscious strategies in the past 12 months, confidence in using these strategies, delayed and forgone care owing to cost, health service utilization, health insurance literacy, health literacy, technological access, and sociodemographic variables. Survey measures on participants’ previous use of and confidence in using cost-conscious strategies were derived from a nationally representative survey of HDHP enrollees conducted by Kullgren et al [12], which included these items after extensive cognitive interviews. Confidence in using cost-conscious strategies was measured on a range of 4 to 40, a sum of 1 to 10 scores for each of the 4 strategies: comparing costs, comparing quality, discussing costs with providers, and putting aside money for care. We used the 21-item Health Insurance Literacy Measure, with health insurance literacy measured on a scale of 21 to 84 (high scores indicate high health insurance literacy) [23]. We also used the single health literacy screening question by Chey et al [24], in which low health literacy was defined as being “somewhat” or less confident in completing medical forms independently. Survey items about technological access were derived from the Health Information National Trends Survey [25]. These survey measures were chosen because they were the factors that we hypothesized would most strongly influence participants’ intervention preferences.

Descriptive statistics were used to assess the characteristics of the interview and survey participants. Survey data captured the frequencies of interest in each type of potential educational content and potential delivery modalities. Quantitative data analysis was conducted using Stata (version 16.0).

## Results

### Participant Characteristics

Table 2 shows the characteristics of both interview (n=20) and survey (n=432) participants. The median age of the interview participants was 50 (IQR 24.5) years. Interview participants were primarily White (16/20, 80%), had at least a college degree (17/20, 85%), and had an annual household income between US $50,000 and US $99,999 (9/20, 45%). Overall, one-fifth (4/20, 20%) of the interview participants were enrolled in an HDHP through a health insurance exchange, with the remaining 80% (16/20) enrolled in employer-sponsored HDHPs.

**Table 2:** Characteristics of interview and survey participants.

Characteristics		Interview participants (n=20)	Survey participants (n=432)
**Age (years), n (%)**			
	18-35	5 (25)	90 (20.8)
	36-51	7 (35)	153 (35.4)
	52-64	8 (40)	189 (43.8)
Gender (women), n (%)		12 (60)	189 (43.8)
**Race and ethnicity (all that apply), n (%)**			
	White	16 (80)	364 (84.3)
	Black	1 (5)	31 (7.2)
	Asian	2 (10)	28 (6.5)
	Hispanic	2 (10)	23 (5.3)
	Other	0 (0)	4 (0.9)
**Education, n (%)**			
	Some college or less	3 (15)	131 (30.3)
	College degree	5 (25)	159 (36.8)
	Graduate or professional school	12 (60)	142 (32.9)
**Annual household income (US $), n (%)^a^**			
	<50,000	5 (25)	81 (18.8)
	50,000-99,999	9 (45)	141 (32.6)
	≥100,000	6 (30)	201 (46.5)
Enrolled in exchange health plan, n (%)		4 (20)	62 (14.4)
Health insurance literacy score, median (IQR)^b^		60.5 (13.5)	68 (21)
Low health literacy, n (%)^c^		4 (20)	62 (14.4)
Confidence in using cost-conscious strategies, median (IQR)^d^		20.5 (9.5)	31 (12)
**Chronic conditions, n (%)**			
	Diabetes	2 (10)	221 (51.2)
	Hypertension	11 (55)	187 (43.3)
	Asthma	9 (45)	135 (31.3)
	Coronary artery disease	0 (0)	6 (1.4)
	Chronic obstructive pulmonary disease	2 (10)	20 (4.6)
Has >1 chronic condition, n (%)		3 (15)	122 (28.2)

^a^Of the 432 survey participants, 9 (2.1%) did not complete the measure of their annual household income.

^b^Health insurance literacy score ranges from 21 to 84, summed across 21 questions [23].

^c^Low health literacy was defined as being “somewhat” or less confident in completing medical forms [24].

^d^Confidence in using cost-conscious strategies was measured on a range of 4 to 40, summed across 4 questions.

The median age of survey respondents was 47 (IQR 19) years. Survey participants were also mostly White (364/432, 84.3%), and most had graduated from college (301/432, 69.7%). Approximately half (201/432, 46.5%) of them reported an annual household income of >US $100,000. Less than one-fifth (62/432, 14.4%) of the participants had an exchange health insurance plan.

The median health insurance literacy scores were 60.5 (IQR 13.5) in the interview sample and 68 (IQR 21) in the survey sample. Interview participants reported low levels of confidence in their ability to engage in cost-conscious strategies (median 20.5, IQR 9.5) compared with survey participants (median 31, IQR 12).

### Interview Themes

#### Overview

Interviews yielded four main themes related to health care experiences and intervention preferences: (1) financial and access challenges, (2) promising cost-conscious strategies, (3) barriers to engaging in cost-conscious strategies, and (4) key intervention preferences for an intervention to support engagement in cost-conscious strategies. Tables 3 and 4 present illustrative quotes for each of these themes.

**Table 3:** Interview themes and illustrative quotes related to challenges of HDHPs^a^.

Categories under the theme—financial and access challenges in HDHP	Quotations
Delayed or forgone care	“I do think a little bit before a doctor’s appointment and maybe I let things go a little longer than I would because it’s costly.” [Woman aged 62 years, with hypertension and COPD^b^]
Difficulty in affording high deductibles and copayments	“For me, that’s been something that’s been difficult to get used to is paying everything out of pocket, right upfront.” [Woman aged 25 years, with hypertension] “I couldn’t predict that I would have an emergency and have to be hospitalized so soon in January, that it would hit the deductible twice...that I would have that many bills.” [Woman aged 62 years, with hypertension and COPD] “It’s kind of overwhelming. Because it feels like you don’t have insurance. It’s just paying everything out of pocket.” [Woman aged 49 years, with hypertension]
Difficulty in managing chronic conditions in HDHP	“It’s been challenging because medicines are so inflated. A daily inhaler is over $500. Sometimes you ask yourself, you struggle with whether to get a refill today or next month...You don’t always get your medicines. You have to make a decision.” [Woman aged 62 years, with hypertension, diabetes, and COPD] “There was a time when I couldn’t afford my inhaler because it was just too expensive.” [Woman aged 39 years, with asthma]
Difficulty in anticipating out-of-pocket costs for health services	“You have this high deductible, but even after you hit the deductible...we still have co-insurance on several services...It makes it hard...to understand exactly how much we’re going to be paying for the year.” [Man aged 45 years, with hypertension] “Going into a doctor’s visit or lab visit or going in to get a prescription, I’m not exactly sure how much something will cost and how much it will contribute towards the deductible and the out of pocket spending limits, and I only kind of know that after the fact.” [Man aged 25 years, with asthma]

^a^HDHP: high-deductible health plan.

^b^COPD: chronic obstructive pulmonary disease.

**Table 4:** Interview themes and illustrative quotes related to participants’ perceptions of cost-conscious strategies and intervention preferences.

Themes and categories			Quotations
**Promising cost-conscious strategies**			
	Discussing costs with providers to prioritize care	“I will try and do a less expensive route. I will say if the doctor says, let’s have a CT scan to see if something’s going on, I’ll say, can it wait, can we wait a week to see if the symptoms subside?” [Woman aged 62 years, with COPD^a^ and hypertension]	
	Saving for health expenses	“Yes...we use a health savings account. So, when we do have medical needs, we’re able to fund it through that and tax...There’s always money there for medical care.” [Man aged 64 years, with hypertension]	
	Comparing costs	“I thought [GoodRx] was a really useful site where I could actually see, okay if I go to Kroger, it will cost this much. If I go to Rite Aid or CVS, it will cost this much, and I was able to kind of make decisions given that information.” [Man aged 25 years, with asthma]	
**Barriers to engagement in cost-conscious strategies**			
	Difficulty in knowing whom to talk to about costs	“I know doctors don’t want to talk about money at all so it’s really the office staff who can help, and then they say, ‘Call insurance’...My insurance company has not been very easy to work with when I called them with questions...” [Woman aged 39 years, with hypertension]	
	Not enough time and undue hassle	“Sometimes it feels like it’s just too much of a hassle to find that information, and I wonder if I end up paying more than I need to, because I don’t have the time to go to multiple different websites...and figure out what would be the cheapest option.” [Woman aged 31 years, with asthma]	
	Inability to save for health expenses	“I know people are going to find it hard to afford it. But then they say, “Well you have these savings plans.” Well, these savings plans, they cost money too.” [Woman aged 58 years, with asthma]	
	Limited knowledge about cost-conscious strategies	“I have not [compared prices] because to be honest with you, I didn’t know you could even do that.” [Woman aged 25 years, with hypertension] “I don’t really understand the difference between the types of plans, HMO, HSA, PPO...You can obviously save more money if you get a different type of plan, but I don’t know how to maximize that. So I wish I had a better understanding of that.” [Woman aged 32 years, with asthma]	
**Key intervention preferences**			
	Content—interest in learning to anticipate and compare prices	“I think real time and local estimates of the cost of health care would be wonderful. Like what typical hospital charges are for an emergency room visit. What typical lab costs might be...What I can expect in terms of medication costs...estimates that would help us plan for our deductible.” [Man aged 45 years, with hypertension]	
	Modality—interest in web-based resource	“I’d want something that I’d have access to online or on my smartphone. But I can also then search for as well to narrow down what I was looking at and looking for.” [Man aged 35 years, with asthma]	
	Frequency of intervention use	“On-demand would probably be the most useful...available on-demand, but monthly reminders that you have access to it.” [Man aged 64 years, with hypertension]	

^a^COPD: chronic obstructive pulmonary disease.

#### Financial and Access Challenges

Within the *financial and access challenges* theme, participants reported difficulties they faced in accessing needed care for their chronic conditions in their HDHPs (Table 3). Of the 20 interviewees, 15 (75%) reported difficulty in affording health care and managing their OOP spending. Many participants had delayed or forgone needed care owing to cost, had difficulty in affording high cost sharing, and were unable to anticipate OOP costs for services to manage their chronic conditions.

#### Experiences With Cost-Conscious Strategies

Promising cost-conscious strategies were identified through participants’ previous experiences in managing their high deductibles. When asked about their use of cost-conscious strategies, of the 20 interviewees, 12 (60%) interviewees had saved for health care expenses, 10 (50%) had discussed costs with their provider, and 7 (35%) had compared prices using the web. Most (18/20, 90%) of those who used these strategies found them helpful for managing their health care costs. As illustrated in Table 4, participants were better able to manage their spending and make informed health care decisions by talking with providers to strategize their care, saving for anticipated health expenses in HSAs or FSAs, and comparing costs at different pharmacies.

More than half (11/20, 55%) of the interviewees faced barriers to engaging in cost-conscious strategies, including low confidence in using these strategies; difficulty in finding cost information; loyalty to providers with whom they had an established relationship; and limited knowledge of how to navigate HSAs, FSAs, and price comparison tools.

#### Key Intervention Preferences

Interviewees were most interested in a website or app-based intervention that would allow them to easily search for information when they needed to plan for or seek health care. Many participants expressed interest in an intervention that would help them better understand health insurance terms and find transparent OOP cost estimates to manage spending in their HDHPs (Table 4). When asked about their preferences for informational content, more than half (13/20, 65%) of the participants said that they would use an intervention to help them compare prices, and some expressed interest in comparing quality at different locations. Several participants suggested that an intervention should include strategies that had helped them save on their own health care costs (eg, initiating conversations about cost with providers, saving in HSAs or FSAs, and comparing prices) to benefit other HDHP enrollees with chronic conditions. Other suggested intervention content included information about where to find exact insurance coverage information to avoid surprise billing, tools to seek low-cost alternatives for health services, and affordable care options for those who are unable to save for needed care.

### Survey Analysis

#### Financial and Access Challenges

Most (292/432, 67.6%) survey participants had been enrolled in their HDHP for at least 2 years, and most (255/432, 59%) had met their deductible in 2020. In the past 12 months, approximately one-third (147/432, 34%) of them reported having been hospitalized at least once, and a similar percentage (160/432, 37%) reported having at least one emergency room visit. More than one-third (170/432, 39.4%) of the participants reported delayed or forgone care owing to cost in the past 12 months.

#### Experiences With Cost-Conscious Strategies

In the past 12 months, most (298/432, 68.9%) participants had put aside money for anticipated health care costs, approximately half had engaged in conversations about cost with a clinician (230/432, 53.2%) and compared quality of care at different locations (223/432, 51.6%), and less than half of them had compared prices for health services (187/432, 43.3%). Most (373/432, 86.3%) of them had used technological devices to search for health information, and most (265/432, 61.3%) had used devices to track health care costs.

#### Key Intervention Preferences

**Figure 2:**
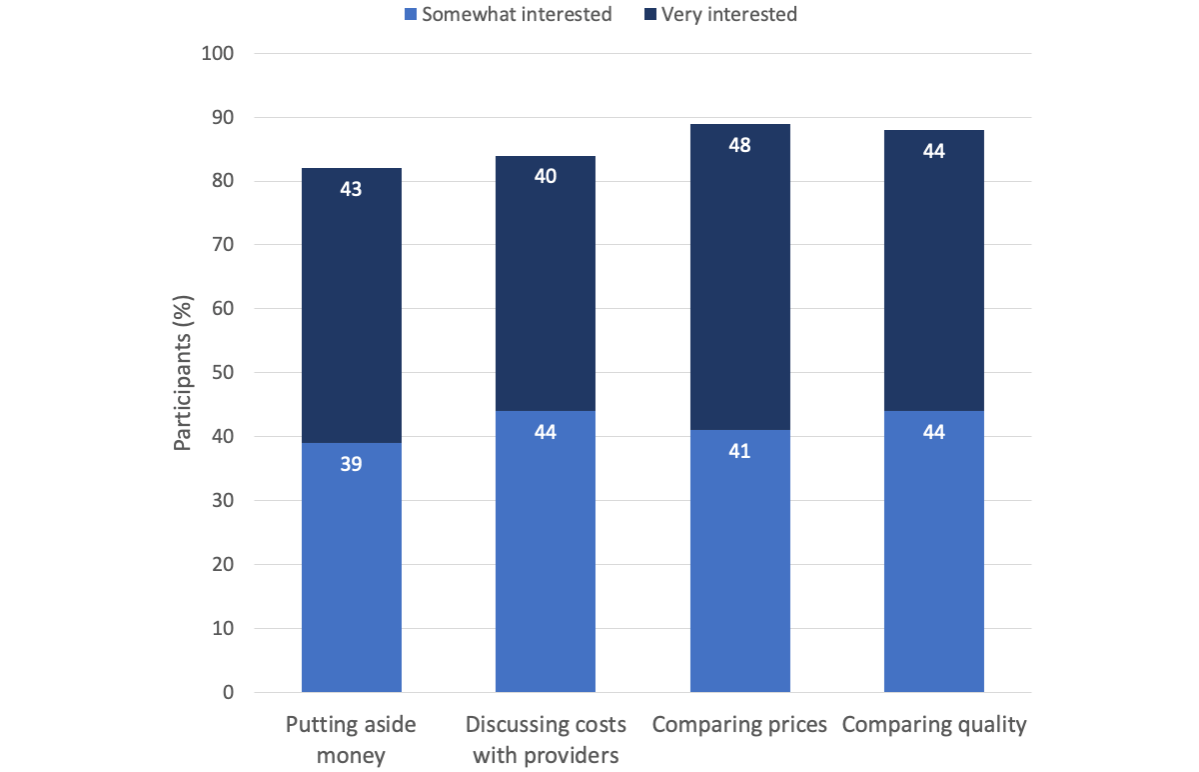
Survey participants’ preferences for intervention content.

**Figure 3:**
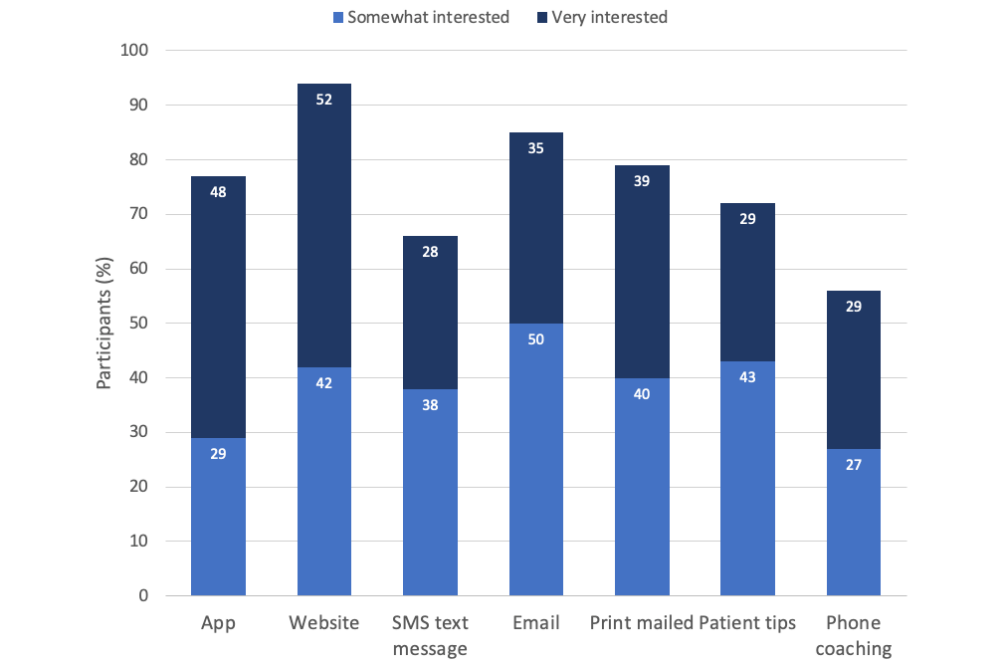
Survey participants’ preferences for intervention modality.

As displayed in Figure 2, participants were most interested in learning to compare prices (387/432, 89.6% were somewhat or very interested), followed by learning to compare quality (381/432, 88.2% were somewhat or very interested), discussing costs with providers (364/432, 84.3% were somewhat or very interested), and putting aside money for health services (355/432, 82.2% were somewhat or very interested). Figure 3 shows the level of interest in receiving this information through different types of potential modalities. Most participants were somewhat or very interested in a website (408/432, 94.4%), followed by email (368/432, 85.2%), print mailings (342/432, 79.2%), and an app (333/432, 77.1%). A plurality of participants (196/432, 45.4%) said that they would want to engage with such an intervention a few times in a year, 21.9% (95/432) said that they would use it monthly, and 12.5% (54/432) said that they would use it weekly.

## Discussion

### Principal Findings

More than half (18/20, 90%) of HDHP enrollees with chronic conditions who participated in this mixed methods study had saved for health care costs and talked to their providers about cost, but few of them had experience with comparing prices for, and quality of, services at different locations. On the basis of interview responses, comparing prices and discussing costs with providers were the most challenging strategies because of the difficulty in finding transparent information about health care costs and knowing whom to ask for cost information. Half (10/20, 50%) of the interviewees had had positive experiences when discussing costs with providers because they were able to obtain coupons, postpone elective services, and consider alternative treatment options or low-cost facilities. However, some interviewees were skeptical about the benefit of discussions with their providers, citing experiences in which a provider had given them limited or incorrect information about their OOP costs. Many interview participants also expressed interest in comparing prices for services such as procedures, laboratory tests, imaging, and prescription drugs but were deterred by loyalty to the providers and health systems with whom they had established care. Most of the interviewed (17/20, 85%) and surveyed (406/432, 93.9%) participants were interested in a web-based informational intervention that they would use a few times in a year to help them find and use web-based price and quality comparison tools, learn to discuss costs with providers, and save for medical expenses.

### Limitations

This study has several limitations. Our samples included few racial and ethnic minorities, and most (17/20, 85% of the interview participants and 301/432, 69.7% of the survey participants) participants had at least a college degree. Although individuals in our samples had relatively high incomes, education, and health literacy, they still faced considerable financial challenges when seeking health care (eg, 173/432, 40% of the survey participants delayed or went without needed care owing to cost in the past year). This suggests an even more pressing need for interventions to support individuals with lower income, health literacy, and formal education. The preferences among our highly educated participants may also differ from those of HDHP enrollees with low levels of education and health insurance literacy, who may benefit more from such an intervention. Our survey sample was not nationally representative because it was corroborated by qualitative interview data, and we felt that a nonrepresentative sample would be sufficient to gather a nationwide range of experiences and perspectives across this patient population. Relatively few individuals with coronary artery disease participated in the study. Many survey items were newly created for this study and have not undergone formal reliability testing; however, they were created based on qualitative themes that emerged during interviews. The use of a 3-point scale to assess interest in intervention components may have limited our precision in measuring intervention preferences. While analyzing the survey results, our focus was on quantifying participants’ preferences, and thus, we used bivariate analyses rather than multivariable analyses. Future studies could more closely examine the variation in perspectives across different subgroups and also include patients with other relatively common chronic conditions such as mental health conditions, which can also lead to high OOP spending in HDHPs.

The demographic data we collected from participants did not include household size, and thus, we were unable to calculate household income as a percentage of the federal poverty level. Finally, interview participants were recruited through U-M Health Research [22] after completing a series of eligibility screening questions about their deductible amounts, insurance source, and confidence in engaging in cost-conscious strategies. The confidence measure was subjective, and participants who chose to complete a screening questionnaire on health insurance may have been more knowledgeable about their HDHPs than those who chose to not complete the screening process.

### Comparison With Previous Studies

Owing to high cost sharing in HDHPs, many enrollees delay and forgo necessary care [4], experience confusion regarding covered services, and feel unable to control costs at the point of care [3]. A national survey of HDHP enrollees found that patients enrolled in HDHPs could decrease costs by being more cost-conscious health care consumers; however, few HDHP enrollees use cost-conscious strategies, largely because they had not considered it [12]. This study applied the Health Belief Model to identify intervention components that would help to raise patients’ self-efficacy in using cost-conscious strategies.

Previous interventions have focused on promoting conversations between patients and physicians [26], helping patients understand health insurance terms [27] and compare prices at different locations [28] and facilitating self-management of complex chronic conditions [29]. However, most existing resources stop short of helping patients be more engaged and informed health care consumers as a strategy to help them better afford needed care. The Choosing Wisely campaign [30], for example, guides patients through navigating conversations with their providers about health care choices, but not about cost. Although price comparison tools such as Healthcare Bluebook [28] and other third-party and health plan applications exist, few patients realize that these tools are available [31], and many are unsure about how to use them [18,32]. Therefore, these applications have had little impact on patients’ ability to afford needed health care. In a study, for example, when employees were offered a price transparency tool, only 10% of them used it, and the mean OOP spending increased by US $18 [17].

A recent rule by the Centers for Medicare and Medicaid Services requires hospitals, as of January 2, 2021, to publish consumer-friendly lists of negotiated rates for 300 *shoppable services* [33,34]. Beginning in 2023, health plans will similarly be required to offer a web-based shopping tool for consumers to see negotiated rates and OOP cost estimates for 500 shoppable services [35]. Hospital compliance with these regulations has been inconsistent [36]. Our findings suggest that patients are interested in comparing prices but are less confident in using price information owing to lack of knowledge. Given that our participants had relatively high levels of education and health insurance literacy but still often lacked confidence in navigating price transparency tools, there is an even more critical need for helping those with lower levels of education and health insurance literacy to use cost-conscious strategies and navigate existing tools. Although some participants reported being hesitant to shop for care because of loyalty to a particular health system, interventions could be tailored to compare prices and quality for outpatient procedures, physical therapy, laboratory tests, and imaging services—these are often costly and nonurgent, and they rely less on longitudinal patient-provider relationships [18]. Patients with chronic conditions in HDHPs could especially benefit from additional education on cost-conscious strategies, as our interviews found that these patients often face high cost sharing burdens [10] and are unsure about which services are subject to their deductibles [7].

Survey and interview participants also expressed interest in learning how to effectively discuss costs with their providers. In addition to patient-facing interventions to help patients initiate cost conversations with their providers, future studies and operational initiatives could examine provider-facing technological solutions that integrate cost information into electronic health records, so that providers have information at the point of care to facilitate high-value conversations and decision-making [37]. Schiavoni et al [38] found that primary care physicians, when presented with patient-specific price information, were more likely to engage in conversations with patients to seek more affordable treatment options. Current real-time benefit tools, which display patient-specific OOP costs when a provider orders a medication, have been shown to generate savings for patients but only display lower-cost alternatives for a small proportion of prescriptions [39]. Such tools embedded into the provider’s workflow could be expanded to cover other costly services and further encourage patient-physician cost conversations.

### Conclusions

These results suggest that some of the cost-related challenges that HDHP enrollees with chronic conditions face in their health plans could be mitigated by an intervention that supports their use of cost-conscious strategies when planning for and seeking health care. On the basis of these findings, we are in the process of developing and pilot-testing a website-based and email-based intervention to teach HDHP enrollees with chronic conditions to better understand and use their health plans.

Our results suggest that future interventions should be delivered through an accessible, web-based modality with effective cues to use cost-conscious strategies. To improve affordability for this patient population, future interventions should also ensure high utility for those with multiple chronic conditions and low health insurance literacy, who may face great risks of high OOP spending in HDHPs. As many participants reported inaccessibility of transparent price information to be the biggest barrier, interventions and policies should focus on improving the accessibility of price transparency tools to mitigate this challenge. As enrollment in HDHPs continues to increase, health systems, health plans, and employers should explore these short-term strategies and advocate for long-term policy changes to better support the growing number of Americans who are facing high cost sharing in these plans.

## Appendix

Multimedia Appendix 1 Supplemental interview guide and survey instrument.

## Abbreviations

FSA: flexible spending account
HDHP: high-deductible health plan
HSA: health savings account
OOP: out-of-pocket
U-M: University of Michigan
