# Supplementary material for: Helping Patients With Chronic Conditions Overcome Challenges of High-Deductible Health Plans: Mixed Methods Study
Source: JMIR Form Res. 2023 Jan 31;7:e37596. doi: 10.2196/37596 (PMC9929725; doi:10.2196/37596)
Supplement: Multimedia Appendix 1 [file formative_v7i1e37596_app1.docx]

**Supplemental Interview Guide 1**

*Hello, this is [name of interviewer(s)] from the University of Michigan. Thank you for agreeing to be interviewed for our study. Before we begin, I would like to go over the consent document to obtain your verbal consent to participate in the study.*

*[Go over consent document]*

*I’d like to audio-record the interview and it will last about 30 minutes. Does that work for you?*

*Please let me know if at any time you want to take a break. Also, if for any reason you do not want to answer any of the questions, just let me know and I will skip that question.*

*Do you have any questions or concerns before we begin recording? [If no] I’ll start the recorder now. [After starting recorder] And to confirm, do you agree to me audio-recording our interview? As a reminder, all your responses will be made anonymous and kept confidential.*

*[If no consent to audio-recording]: Okay, then we will proceed without audiotaping and take notes by hand.*

*Before we begin, do you have any other questions for me?*

1. First, I would like to ask about your health in general. You recently told us that you have the following chronic health condition/s: [STATE CHRONIC CONDITIONS FROM SCREENING QUESTIONNAIRE].
   1. How long have you had these condition/s?
   2. What types of health care have you needed for this condition/these conditions (e.g., hospitalizations, visits to a doctor or other health care professionals, lab tests, imaging tests, medications)? About how often do you need these?
   3. How have these conditions affected your life (e.g., financially, quality of life, day-to-day activities)?
2. Next, I would like to talk about your health insurance. You had previously told us that your health insurance deductible -- the amount you pay for covered health care services before your insurance plan starts to pay -- is [STATE DEDUCTIBLE AMOUNT FROM SCREENING QUESTIONNAIRE], and that you acquired your health insurance from [STATE COVERAGE SOURCE FROM SCREENING QUESTIONNAIRE].
   1. How long have you been enrolled in this insurance plan?
      1. If enrolled >1 year: Did you meet your deductible in 2019?
      2. Have you already met your deductible for 2020?
      3. How much do you think you'll spend on your health care this year?
   2. Who chose your insurance plan (e.g., you or someone else in your household)? What factors did you (or they) consider when choosing your insurance plan (e.g., premiums, health savings account opportunities, health conditions, incentives from an employer)? What sources of information did you/they use in making this decision?
   3. What about your insurance plan makes it easier for you to get the care you need for your chronic conditions (e.g., the network of providers)?
   4. What about your insurance plan makes it harder for you to get the care you need for your chronic conditions?
   5. Have you considered changing your current insurance plan? If so, why?
   6. What do you wish you had known before you had enrolled in your current insurance plan?
   7. What do you wish your health care providers knew about your insurance plan?
3. With your current insurance plan, what strategies, if any, have you used to manage your health care costs?
   1. Probe 1: Have you talked with your health care providers about your health care costs? If so, how has that worked for you? What could have made that more helpful? If not, why not?
   2. Probe 2: Some people use websites or smart phone apps to check prices for health care at different places. Is this something you have done? If so, which ones did you use? How has that worked for you? What could have made that more helpful? If not, why not?
   3. Probe 3: Some people use special saving accounts, such as a Health Savings Account (HSA) or Flexible Spending Account (FSA), to save for health care. Is this something you have done? If so, how has that worked for you? What could have made that more helpful? If not, is this something you have heard of? Do you have such accounts available to you? If so, why have you not used them?
4. Our research team is developing an informational program to help people like you --people who have a chronic condition and are enrolled in an insurance plan with a high deductible -- manage their health care costs.
   1. What do you think of this idea in general?
   2. What types of information would be most helpful for the program to provide?
      1. Probe 1: How about information on how to communicate with health care professionals and staff about your health care expenses? What would you want to know?
      2. Probe 2: How about information on how to find and use health care prices to plan for costs of health care you will need in the future? What would you want to know?
      3. Probe 3: What about information on how to use health care prices to choose where to seek health care? What would you want to know?
   3. How would you want to receive information from this program (e.g., paper, website, smartphone app)?
   4. How often would you want to receive information from this program (e.g., daily, weekly, monthly)?
   5. Would it be better if this information were sent to you routinely (e.g., every week or every month), or only when you are in the process of accessing health care?
   6. Beyond what we just talked about, what else should this informational program include to help people with chronic conditions who have a high-deductible health plan manage their health care costs?

*Thank you very much for taking the time to talk with me today.* Do you have any questions for me? I have a survey to go over with you, and it should it not take more than two minutes to complete.

**Supplemental Survey Instrument 2**

Thank you for your interest in our survey on health insurance! First, we have a few questions to see if you are eligible to complete this survey.

SCREENING QUESTIONS

1. Do you have any kind of health insurance that covers all or some of your costs when you use health care services (e.g., private insurance, Medicaid, Medicare, VA, etc.)?
   1. Yes
   2. No

***[If ScreenQ1 = B or refused, terminate and insert standard close]***

1. Which of the following is your primary source of health insurance coverage?
   1. Health insurance provided through your or someone else’s employer or union
   2. Medicaid or another state medical assistance plan for people with lower incomes
   3. Health insurance you bought through a state or federal marketplace/exchange under the Affordable Care Act (ACA)
   4. Medicare, a government program for people 65 or older and for some people with certain conditions
   5. Veterans Affairs (VA), Department of Defense, or other military programs
   6. Health insurance from some other source
   7. I do not have any health insurance/coverage

***[If ScreenQ2 = A or D, continue, or else terminate and insert standard close]***

1. Are you the only person who is covered under your health insurance plan or is there at least one other person?
   1. Just me
   2. Me and at least one other person
2. A deductible is the amount of money you have to pay each year before your health insurance will start paying for most health care services. How much is the annual deductible for medical care for your plan?

When answering this question do not think about any separate deductibles you might have for prescription drugs, hospitalization, or out-of-network care.

- 1. Less than [**if ScreenQ3=A:** $1,400 **/ if ScreenQ3=B**: $2,800**]**
  2. **[if ScreenQ3=A:** $1,400 **/ if ScreenQ3=B:** $2,800**]** or more
  3. Do not know

***[If ScreenQ4 = B, continue, or else terminate and insert standard close]***

1. In 2020, were you enrolled in a health plan with a deductible of **[if ScreenQ3=A:** $1,400 **/ if ScreenQ3=B:** $2,800**]** or more?
   1. Yes
   2. No

***[If ScreenQ5 = A, continue, or else terminate and insert standard close]***

1. Have YOU been diagnosed by a doctor or other qualified health professional with any of the following medical conditions? (Select all that apply.)
   1. Diabetes
   2. Hypertension
   3. Asthma
   4. Coronary artery disease (CAD)
   5. Chronic obstructive pulmonary disease (COPD)
   6. None of the above

***[If* Screen*Q6 = refused or none selected, terminate and insert standard close]***

**Standard close:**

Thank you for answering these questions. Based on your responses, you are unfortunately not eligible to complete this survey. Thank you for your time!

**Standard intro:**

Based on your previous responses, you are eligible to complete this survey. It will take about 10-15 minutes to complete. Taking this survey is voluntary, and you can skip any questions that you don’t want to answer. Clicking [“Next”] below means that you agree to take the survey.

If you have any questions about this study, please do not hesitate to contact our Research Coordinator, Iman Ali, at imanali@med.umich.edu.

SURVEY

Health Insurance Plan

First, we have some questions about your health insurance plan.

1. How long have you been in a health insurance plan with a deductible of **[if ScreenQ3=A:** $1,400 **/ if ScreenQ3=B:** $2,800**]** or more?
   1. Less than 1 year
   2. 1-2 years
   3. More than 2 years
2. Did you meet your deductible last year (i.e., in 2020)?
   1. Yes
   2. No
   3. Not sure

Health Insurance Literacy

This next set of questions is about health insurance in general.

1. When choosing a health insurance plan, how confident are you that…
   1. You understand health insurance terms?
   2. You know where to find the information you need to choose a health plan if you were not offered insurance through an employer?
   3. You know how to estimate what you have to pay for your health care needs in the next year, not including emergencies?
   4. You know where to go for help if you were having trouble affording health insurance outside an employer?
   5. You know what questions to ask so you can choose the best health plan for you?
   6. You would choose the health plan that is best for you?

**Across:**

1. Not at all confident
2. Slightly confident
3. Moderately confident
4. Very confident
5. When comparing health insurance plans, how likely are you to…
   1. Understand how the plans differ?
   2. Find out if you have to meet a deductible for health care services?
   3. Look to see which doctors and hospitals are covered in each plan?
   4. Understand what you have to pay for prescription drugs?
   5. Understand what you would have to pay for emergency department visits?
   6. Understand what you would have to pay for specialist visits?
   7. Find out if the plans cover unexpected costs such as hospital stays?

**Across**:

1. Not at all likely
2. Somewhat likely
3. Moderately likely
4. Very likely
5. When using health insurance, how confident are you that…
   1. You know how to find out what is and is not covered before you receive a health care service?
   2. You know how to figure out your share of the cost for care, after the health plan pays their share?
   3. You know what questions to ask your health plan if you have a coverage problem?
   4. You know what to do if your health plan refuses to pay for a service you think should be covered?

**Across**:

1. Not at all confident
2. Slightly confident
3. Moderately confident
4. Very confident
5. When using your health insurance plan, how likely are you to…
   1. Look into what your health plan will and will not cover before you get health care services?
   2. Look to member services to tell you what medical services your health plan covers?
   3. Find out if a provider is in-network before you see him/her?
   4. Review the statements you get from your health plan showing what you owe and what they paid for a service?

**Across**:

1. Not at all likely
2. Somewhat likely
3. Moderately likely
4. Very likely

Health Care Utilization

Next, we have some questions about your use of health care services. As you think about these questions, think only about your own individual health care and **not** health care that was for someone else. Your best estimate is fine.

1. During the past 12 months…
   1. How many times did you see a doctor or other health care professional about your own health at a doctor’s office or clinic, or as a telehealth visit? Do not include times you were hospitalized overnight, visits to hospital emergency rooms, dental visits, or telephone calls.
   2. How many different times did you stay in any hospital overnight or longer?
   3. How many times did you go to a hospital emergency room about your own health?

**[Integer value]**

1. During the past 12 months…
   1. Have you delayed seeking medical care because of worry about the cost?
   2. Was there any time when you needed medical care, but did not get it because you couldn't afford it?
   3. Did you have problems paying or were unable to pay any medical bills? Include bills for doctors, dentists, hospitals, therapists, medication, equipment, nursing home or home care.

**Down:**

a. Yes

b. No

Health Care Consumer Behaviors

For these questions, please tell us whether you have done the following things while thinking about and getting health care services (e.g., a doctor visit, procedure, test, or medication) for yourself in the past 12 months.

1. In the past 12 months, did you put aside money to pay for any health care services before you needed them (e.g., in a Health Savings Account, Flexible Spending Account, or bank account)?
   1. Yes
   2. No
2. [if yes put aside] In the past 12 months, did you put aside money to pay for health care services in any of the following? (Select all that apply.)
   1. Health Savings Account (HSA)
   2. Flexible Spending Account (FSA)
   3. Personal bank account
3. In the past 12 months, before using a service…
   1. Did you talk with a health care provider (e.g. a doctor, nurse, or pharmacist) about how much any health care services would cost you personally?
   2. Did you compare prices for any health care services at different places?
   3. Did you compare quality ratings for any health care services at different places?

**Down:**

- 1. Yes
  2. No

Confidence (health literacy, confidence in cost-conscious strategies)

This next question is about how you think about your interactions with the health care system.

1. How confident are you filling out medical forms by yourself?
   1. Extremely
   2. Quite a bit
   3. Somewhat
   4. A little bit
   5. Not at all
2. As of right now, how confident are you that you could…
   1. Put aside enough money to pay for health care services before you need them?
   2. Compare prices for health care services at different places?
   3. Compare quality ratings for health care services at different places?
   4. Talk with a health care provider (e.g. a doctor, nurse, or pharmacist) about the cost for health care services?

**[Slider response 1 through 10, 1 = Not at all confident, 10 = Very confident]**

Intervention components/ interest

## Our research team is developing an informational program to help people manage their health care costs. This next set of questions is about what information you would prefer to receive, and how, in such a program.

Interest in Topic/Strategy

1. How interested would you be in learning more about…
   1. How to put aside money to pay for health care services before you need them?
   2. Ways to talk to someone at your doctor’s office about your health care costs?
   3. Strategies for comparing prices for health care services at different places?
   4. Strategies for comparing quality ratings for health care services at different places?

**Across**:

1. Not at all interested
2. Somewhat interested
3. Very interested

Modality Preference

1. How interested would you be in receiving information on these topics through…
   1. An app for your smartphone or tablet?
   2. A website?
   3. Periodic text messages?
   4. Periodic emails?
   5. Print materials mailed to you?
   6. Tips from other patients?
   7. Phone coaching sessions?

**Across**:

1. Not at all interested
2. Somewhat interested
3. Very interested

Frequency of Interaction with Intervention

1. How frequently do you think you would interact with such an informational program?
2. Never
3. Once a year
4. A few times a year
5. Monthly
6. Weekly
7. Daily

Tech Access / Health IT Use

Next, we would like to ask you about your technological devices and internet use.

1. Please indicate if you have any of the following devices. (Select all that apply.)
   1. Tablet (like an iPad, Samsung Galaxy, Motorola Xoom, or Kindle Fire)
   2. Smartphone (like an iPhone, Android, Blackberry, or Windows phone)
   3. Basic cell phone
2. How often do you access the Internet through a computer (i.e., a desktop or laptop)?
   1. Daily
   2. Weekly
   3. Monthly
   4. A few times a year
   5. Never
3. How often do you access the Internet through a mobile device (such as a smartphone or tablet computer)?
   1. Daily
   2. Weekly
   3. Monthly
   4. A few times a year
   5. Never
4. In the past 12 months, have you used a computer, smartphone, or other electronic means to look for health or medical information for yourself?
   1. Yes
   2. No
5. In the past 12 months, have you used a computer, smartphone, or other electronic means to track your health care costs (e.g., through an app)?
   1. Yes
   2. No

Demographics

This final set of questions asks for some information about you and your background. This information will remain confidential. You do not have to answer any questions that you feel do not feel comfortable answering.

1. What is your age?
2. What is your gender?
   1. Male
   2. Female
   3. Transgender male
   4. Transgender female
   5. A gender not listed here ______
   6. No answer
3. What is your race and ethnicity? (Select all that apply.)
   1. White
   2. Hispanic, Latino, or Spanish
   3. Black or African American
   4. Asian or Asian American
   5. American Indian or Alaska Native
   6. Middle Eastern or North African
   7. Native Hawaiian or Other Pacific Islander
   8. Some other race or ethnicity
4. What is the highest grade or level of schooling and/or training you completed?
   1. Less than 8 years
   2. 8-11 years
   3. 12 years or completed high school or GED
   4. Vocational, technical, or business training
   5. 1-3 years of college/ junior/ community college
   6. 4 or more years of college or graduated from college
   7. Graduate or professional school
   8. Other (please specify):
5. How many individuals, including yourself, live in your household?
6. What was your total household income before taxes during the past 12 months?
   1. Less than $25,000
   2. $25,000 to $49,999
   3. $50,000 to $74,999
   4. $75,000 to $99,999
   5. $100,000 to $149,999
   6. $150,000 or more
   7. I don’t want to answer this question
7. Last week, did you do any work for pay at a job (or business)?
   1. Yes
   2. No
8. In what region of the US do you currently live?
   1. West
   2. Midwest
   3. South
   4. Northeast

**Survey Close**: Thank you very much for completing our survey! Your answers will help us develop a program to help people better manage health care costs. If you have any questions, you can contact our Research Coordinator, Iman Ali, at imanali@med.umich.edu.
